# Resilience-Informed Community Violence Prevention and Community Organizing Strategies for Implementation: Protocol for a Hybrid Type 1 Implementation-Effectiveness Trial

**DOI:** 10.2196/50444

**Published:** 2023-11-07

**Authors:** Natalie A Blackburn, Stefany Ramos, Michele Dorsainvil, Camara Wooten, Ty A Ridenour, Anna Yaros, Vicki Johnson-Lawrence, Dana Fields-Johnson, Nzinga Khalid, Phillip Graham

**Affiliations:** 1 RTI International Research Triangle Park, NC United States; 2 University of North Carolina School of Medicine Chapel Hill, NC United States; 3 University of Pittsburgh Pittsburgh, PA United States; 4 University of North Carolina Chapel Hill, NC United States; 5 Prevention Institute Oakland, CA United States

**Keywords:** ACEs, community organizing, community violence, implementation-effectiveness trial, resilience

## Abstract

**Background:**

Community violence is a persistent and challenging public health problem. Community violence not only physically affects individuals, but also its effects reverberate to the well-being of families and entire communities. Being exposed to and experiencing violence are adverse community experiences that affect the well-being and health trajectories of both children and adults. In the United States, community violence has historically been addressed through a lens of law enforcement and policing; the impact of this approach on communities has been detrimental and often ignores the strengths and experiences of community members. As such, community-centered approaches to address violence are needed, yet the process to design, implement, and evaluate these approaches is complex. Alternatives to policing responses are increasingly being implemented. However, evidence and implementation guidance for community-level public health approaches remain limited. This study protocol seeks to address community violence through a resilience framework—Adverse Community Experiences and Resilience (ACE|R)—being implemented in a major US city and leveraging a strategy of community organizing to advance community violence prevention.

**Objective:**

The objective of this research is to understand the impact of community-level violence prevention interventions. Furthermore, we aim to describe the strategies of implementation and identify barriers to and facilitators of the approach.

**Methods:**

This study uses a hybrid type 1 effectiveness-implementation design. Part 1 of the study will assess the effectiveness of the ACE|R framework plus community organizing by measuring impacts on violence- and health-related outcomes. To do so, we plan to collect quantitative data on homicides, fatal and nonfatal shootings, hospital visits due to nonaccidental injuries, calls for service, and other violence-related data. In Part 2 of the study, to assess the implementation of ACE|R plus community organizing, we will collect process data on community engagement events, deliver community trainings on community leadership and organizing, and conduct focus groups with key partners about violence and violence prevention programs in Milwaukee.

**Results:**

This project received funding on September 1, 2020. Prospective study data collection began in the fall of 2021 and will continue through the end of 2023. Data analysis is currently underway, and the first results are expected to be submitted for publication in 2024.

**Conclusions:**

Community violence is a public health problem in need of community-centered solutions. Interventions that center community and leverage community organizing show promise in decreasing violence and increasing the well-being of community members. Methods to identify the impact of community-level interventions continue to evolve. Analysis of outcomes beyond violence-specific outcomes, including norms and community beliefs, may help better inform the short-term and proximal impacts of these community-driven approaches. Furthermore, hybrid implementation-effectiveness trials allow for the inevitable contextualization required to disseminate community interventions where communities drive the adaptations and decision-making.

**International Registered Report Identifier (IRRID):**

DERR1-10.2196/50444

## Introduction

Community violence is a persistent problem impacting public health. Community violence impacts the overall mental and physical health of neighborhoods, towns, and collective spaces. Risk factors associated with violence and community trauma include community poverty, community disadvantage, and disorganization [1]. Research framed through social disorganization theory, sometimes referred to as “broken windows theory,” has suggested that disorder within a community can impact mental health and contribute to stress in individuals [2]. Among youths and adolescents, the impacts of community violence are particularly strong [3]. Violence, including experiencing physical violence and being a witness to violence, is considered an adverse childhood experience that can significantly impact children’s future physical and behavioral health [4]. Different types of violence against youths, such as child abuse and neglect and community youth violence, often share the same risk and protective factors [5].

Protective factors, contrasted with risk factors, are increasingly seen as key to effective community-level violence prevention interventions. In taking a strength-based approach, which highlights protective factors, there is an opportunity to center the role of community members and their experiences while moving away from risk-framing approaches [6]. Strength-based approaches to community violence prevention are those that consider community relationships as a positive, protective factor against violence. Resilience theories, along with constructs of collective efficacy and social cohesion, have allowed for an expanded understanding of how to consider structural-level interventions leveraging protective factors to address community violence [7].

Interventions conducted at the community level can impact multiple individuals, particularly youths, at once [8]. Community-level interventions can interrupt patterns of violence at the structural level, preventing exposure to violence at an early age, and can move beyond school-based approaches commonly targeting youth [9]. Furthermore, community-level interventions developed through an asset-based framework, rather than deficits, have gained wider understanding and evidence. One community-level primary prevention strategy that has been explored in the context of community resilience is community organizing [10]. Community organizing can be defined as a process in which people affected by common concerns aim to build their power together and identify solutions for those shared concerns by mobilizing [11,12]. Community organizing, in the context of community violence prevention, seeks to address issues of social cohesion and isolation by building up relationships within communities and having communities lead the violence prevention strategies. Community organizing has been incorporated into interventions seeking to address domestic violence [13], as well as a way to empower youth in the violence prevention space [14,15].

It is difficult to implement community-level interventions and measure their effects, because of the complexity of intermingled contextual and temporal factors. Community-level interventions are often not broadly disseminated nor scaled up because they are developed according to the unique demographic characteristics of the community, as well as the unique characteristics of the practitioners or other persons leading implementation. Community-level interventions may also occur at a particular moment in time that cannot be easily incorporated into statistical models to determine intervention impacts. Hybrid trials seek to address these problems by allowing for implementation-related data collection to be incorporated into the study design [16]. Thus, this study uses a hybrid type 1 implementation-effectiveness trial; these are typically conducted early in the intervention development process and have the primary aim of evaluating the effectiveness of an intervention while allowing for the collection of implementation-related data to improve dissemination and intervention uptake.

Adding to the complexity of researching community-based interventions is the appropriate selection of statistical approaches to analyze community-level outcomes captured as interrupted time series data. Statistical decisions include the choice of analytic model (eg, autoregressive integrated moving average vs regression), if and how to account for autocorrelation and seasonality, if and how to correct for a small sample size, and aligning the statistical strategy with the available data, such as the number of time points. Reporting the planned analytic strategy a priori in protocol papers is important for avoiding bias due to a selected approach, as well as engendering confidence in the results [17].

Promising structural interventions that account for these methodological and implementation issues to address community violence have emerged in recent research. Cure Violence, a violence prevention intervention adapted from “Chicago Ceasefire” that was developed in the mid-1990s, framed violence as a communicable disease [18]. Through adaptations and implementations in multiple cities over the last 3 decades, the impacts of Cure Violence have been mixed [19]. The research has allowed for an evolved understanding of the importance of changing community norms around violence, in addition to the physical acts of violence [20,21]. Another study building on Chicago Ceasefire, the CeaseFire 365 intervention, effectively reduced gun violence in Baltimore while identifying the importance of measuring nonviolence-related outcomes to determine impact. The intervention included weekend “ceasefires” in which community events and public meetings sought to communicate a stop to violence during specific times [22].

Building on previous experiences and challenges of Cure Violence and similar community violence prevention interventions, the Prevention Institute (PI) and the University of California San Francisco, developed the Adverse Community Experiences and Resilience (ACE|R) framework as a community-level approach to violence prevention. The ACE|R framework uses community-specific data combined with an underlying evidence-based understanding of how adverse childhood experiences impact adolescents and communities [10]. In 2016, PI introduced the ACE|R framework to over 150 community partners and residents in Milwaukee during a time when the Milwaukee Blueprint for Peace—a call to action for violence prevention—was in development. The PI partnered with the Milwaukee Office of Violence Prevention (OVP) to facilitate the development of the Milwaukee Blueprint for Peace using the ACE|R framework, including having residents identify the leading risk and resilience factors related to violence in Milwaukee. These factors were solicited through community conversations, roundtable discussions, and web-based surveys. Residents and community organizations shared input on the emerging vision and principles in alignment with the ACE|R framework through those same forums. Through this citywide organizing and mobilization approach to comprehensive planning, a shared set of goals, strategies, and indicators was identified and formally launched in 2017 as the Blueprint for Peace.

The primary objective of this study is to assess whether community organizing and mobilizing residents to act on the Blueprint for Peace increases the effectiveness of the ACE|R framework to reduce community-level measures of child abuse and neglect and youth violence in 4 select communities, compared with having no community organizing in the 6 remaining select communities. The secondary objective is to explore the barriers to and facilitators of implementing community organizing as a strategy for increasing support for the sustainability of the Blueprint for Peace and the reduction of community-level violence in Milwaukee.

## Methods

### Study Design

To meet the objectives of this study, the proposed research design is a hybrid type 1 effectiveness-implementation design using a mixed methods approach, leveraging both quantitative and qualitative methods to measure the effect of the ACE|R plus community organizing on communities and collect data on the implementation of community organizing. We present the details of the proposed study in two parts: part 1 describes the proposed study to measure the effectiveness of the ACE|R plus community organizing, and part 2 describes how we will measure the implementation of the ACE|R plus community organizing.

### Setting

**Figure 1:**
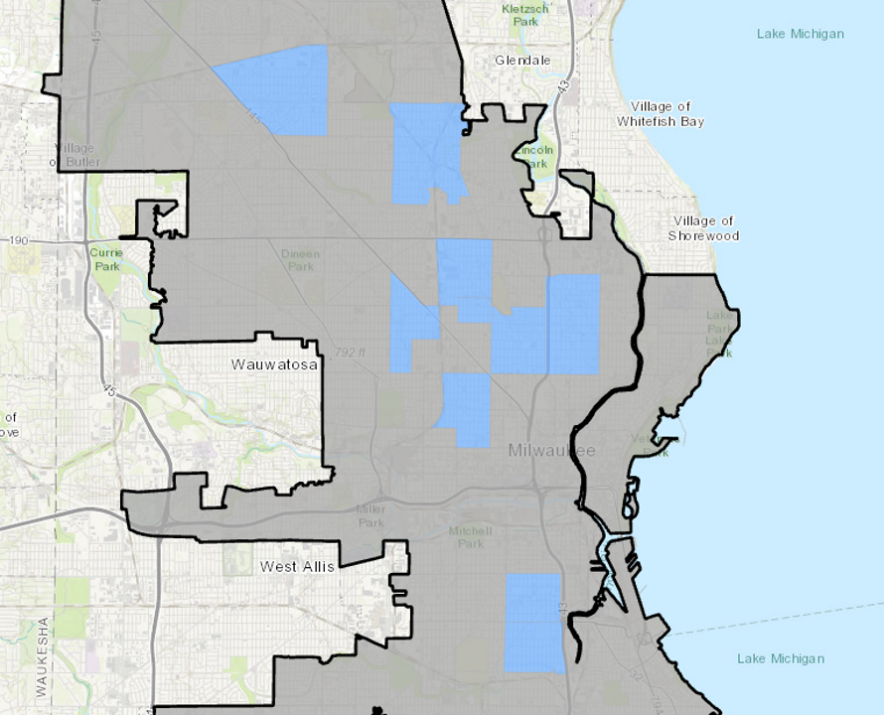
Map of Milwaukee, with the 10 communities where the Blueprint for Peace Initiative has been implemented highlighted in blue.

This proposed study will take place in Milwaukee, Wisconsin, where the Blueprint for Peace was first launched in 2017. Blueprint for Peace participants in the development process selected 10 communities as the neighborhoods to prioritize and focus on violence prevention efforts (Figure 1). This study focuses on 4 of the 10 selected communities, hereafter referred to as the “select communities.”

### Intervention—Blueprint for Peace and the Resident Leadership and Advocacy Training

The proposed study uses the ACE|R framework plus community organizing as a strategy to directly address the need for community-level action to prevent child abuse and neglect and youth violence. Intervention communities will receive training and technical assistance that integrates the ACE|R framework, community organizing and mobilization, and operational knowledge of the Milwaukee Blueprint for Peace**.** The goal of this intervention element is to prepare residents and other interested parties in the community (eg, community-based organizations and faith-based organizations) to understand and be prepared to act on (organize or support) structural community-level change in support of the Blueprint for Peace. The ACE|R plus community organizing trainings (hereafter referred to as “resident leadership and advocacy training”) consist of 5 modules that ground participants in the knowledge of systemic trauma and then cover ways to address neighborhood violence through community-designed solutions. The details of each of the training modules are presented in Table 1.

**Table 1:** Content details for the 5 modules that make up the resident leadership and advocacy training.

Module	Module title	Module objective	Example content
1	Systematic and Historical Trauma (Adverse Community Experiences and Resilience)	Learn about systematic and historical trauma through the recognition that the impact is cumulative and reverberates across neighborhoods for generations	Defines adverse childhood experiences Defines trauma
2	Social Determinants of Health and THRIVE^a^	Explore community factors—people, places, and equitable opportunities—that influence health, safety, and well-being in their neighborhoods	Defines the social determinants of health inequities and describes how socioeconomic factors and neighborhood characteristics shape behaviors Identifies strategies to address community-level factors and move toward health equity Discusses the role of neighborhood, home, and work environments in determining health and the quality of life
3	Addressing and Preventing Community Trauma and Healing	Learn ways to address community trauma through healing	Healing as a starting point for community and system transformation, including activities to explore opportunities for healing and identify strong examples of how community healing is already taking place in the neighborhood Strategies for healing Strategies to address community trauma
4	Building Resident Leadership and Voice, Navigating Systems from Civic Engagement to Movement Building	Identify the steps of effective community action, including needs and issue assessment and strategic planning	Defines key components of a toolkit specific to Milwaukee city and county processes Outlines opportunities for shifting power by elevating community ownership, creating community change, such as shifting policy, and influencing decision makers. An overview of the Blueprint for Peace and the goal areas, including discussion and activities, to map how it is connected to existing neighborhood efforts and neighborhood goals that support the blueprint
5	Resident Leadership in Action—Needs and Opportunity Assessment	Identify actions they will take to implement community action	Readiness assessment designed to guide and support communities in determining capacity-building needs Readiness assessment to build a plan for community mobilization of assets that accelerates community-level violence norms change and collective action toward a public health approach to violence prevention

^a^THRIVE: Tool for Health & Resilience in Vulnerable Environments.

### Part 1: Effectiveness of the ACE|R Framework Plus Community Organizing

To define and determine the effect of a community violence prevention intervention, the first part of this study will be an effectiveness study of the ACE|R framework plus community organizing. This part will include using an idiographic clinical trial research design that captures within-community changes to analyze the Blueprint for Peace–related activities and outcomes, including child abuse and neglect, youth violence, and other violence-related outcomes (Textbox 1).

**Key measures to evaluate the implementation and effectiveness of Adverse Community Experiences and Resilience (ACE|R) plus community organizing through outcome constructs.**

**Outcome constructs and tracked measures**

- Increased community organizing
  Events led by individuals trained in community organizing
- Long-term development of safe and healthy communities
  Homicides
  Nonfatal shootings in select neighborhoods
  Fatal shootings in select neighborhoods
  Firearm-related injuries
  Arrests due to violence among youths younger than 18 years
  Arrests due to child maltreatment
  Calls for service in selected communities
  Nuisance, vacant, and blighted properties abated in select neighborhoods
  Poverty rate in select neighborhoods
- Reduced adverse childhood experiences
  Children hospitalized for confirmed and suspected child abuse or violence-related injury
  Children at the emergency department for confirmed and suspected child abuse or violence-related injury
  Home removals
- Reduced child abuse and neglect
  Child protective services referrals
  Child maltreatment reports
  Rate of child victims per 1000 children
- Increased social cohesion
  Block clubs in select neighborhoods
  Resident-led events in select neighborhoods
- Increased awareness of violence prevention strategies
  Neighborhood perceptions of safety, resilience, and access to resources and services
  Awareness of neighborhood assets, resources, and programming among neighborhood residents

Unlike randomized controlled trials, which use large samples and randomization to control confounding influences, the idiographic clinical trial will manage differences by collecting control data and treatment data from the same community. Differences between control and treatment phases are quantified as change at treatment onset (ie, phase intercepts) and change over time (ie, phase slopes). Blueprint for Peace activities include events, workshops, and trainings that provide communities with strategies and resources to reduce violence. Counts and descriptions of Blueprint activities will be collected in the 4 select communities monthly. The 4 selected communities are defined by geographically bounded areas that city government agencies provided to the research team (Figure 1). Milwaukee is a safe and resilient city where the lives of all residents are valued, promoted, and protected. Outcome measures were derived from the following six overarching goals of the Blueprint for Peace [23], (1) stop the shooting, stop the violence; (2) promote healing and restorative justice; (3) support children, youth, and families; (4) promote economic opportunity; (5) foster safe neighborhoods; and (6) strengthen capacity and coordination of violence prevention efforts.

### Data Analysis

Primary analysis of outcome measures will use intensive hierarchical modeling with maximum likelihood estimation, a multilevel linear model that has evolved for use in small-sample clinical trials [24]. This approach can capitalize on both change from baseline (preintervention) to treatment phases of time series data and differences between communities that do and do not receive an intervention.

Recent developments in intensive hierarchical modeling for small samples allow for evaluation models that (1) control for biases due to small samples, (2) estimate intervention impacts that are quantified in terms of phase “jumps” and slopes, (3) use thorough power analysis using Monte Carlo simulation, and (4) have the ability to model and test complex causal inferences [24,25]. For intensive hierarchical modeling of the ACE|R plus community organizing impacts, time series data are clustered within communities; the effect of community organizing is quantified at level 1 as change over time, with aggregate models across communities being analyzed at level 2. Corrections to statistical testing and parameter estimates (to account for small sample sizes) will include the Satterthwaite degrees of freedom [26] and use full information maximum likelihood estimation for statistical testing of parameters but estimate model parameters using restricted maximum likelihood.

Differences between baseline and treatment phases will be estimated within the 4 select communities; community-level factors that may enhance or attenuate the impact of resident leadership and advocacy training will be explored using between-select communities fixed effects. An additional contrast will be to compare the changes in the 10 select communities to matched communities in Milwaukee outside the study.

### Part 2: Implementation of the ACE|R Framework Plus Community Organizing Strategy

To address the objective of improving the research-to-practice gap, the second part of the study is the collection of data on the implementation of community organizing, as well as neighborhood perceptions of safety, resilience, and access to resources and services. This includes awareness of neighborhood assets, resources, and programming among neighborhood residents. Data sources include community members and resident leaders who have received training on how to deliver training on ACE|R and Blueprint for Peace activities. Data collection activities include focus groups and surveys.

### Recruitment

Recruitment of community residents will occur in collaboration with PI, OVP, and community organizations including neighborhood associations in the 10 select neighborhoods. Materials for recruitment will be cobranded to reach community residents in venues like community centers, food pantries, flea markets, and social service organizations. Social media outreach will take place on the web through Instagram and Facebook. In addition to recruitment in the broader community, those participants who have completed the resident leadership and advocacy training will be referred to the community resident focus groups.

### Focus Groups With Community Members

We plan to conduct focus groups within the select communities every 6 months to better understand the implementation of the Blueprint for Peace and community organizing as a strategy. At least 1 focus group in each select community will occur, with community residents recruited through convenience sampling, resident advisory committees, and local advertisements.

Those who agree to participate in the focus groups will undergo informed consent procedures and receive a US $40 gift card (their choice between Walmart, Amazon, or 2 local Milwaukee vendors) as part of their participation. Focus groups with community participants will be conducted through Zoom or in person in private or semiprivate spaces convenient for the participants (eg, community center) by research staff trained in qualitative methods. Focus groups will be digitally recorded and transcribed verbatim using a third-party service. Once the transcript is returned, it will be autocoded in NVivo (version 12; QSR International). Theme identification will occur for each question on the focus group transcripts.

### Trainings and Community Events

In addition to focus groups with community members, we plan to collect data on the resident leadership and advocacy training conducted by the PI and the events delivered by those trained on resident leadership and advocacy, as well as events hosted by the city of Milwaukee as part of the Blueprint for Peace. Data will be collected through information provided by those trained (through surveys), as well as regular monthly meetings with the Milwaukee OVP.

Guided by the Consolidated Framework for Implementation Research (CFIR), we will use the data from these trainings and community events to better understand key CFIR domains: outer setting, inner setting, intervention characteristics, and process. These data will allow for the definition of specific strategies for improving intervention implementation (Table 2).

**Table 2:** Data sources informing implementation of Adverse Community Experiences and Resilience (ACE|R) plus community organizing through the use of the Consolidated Framework for Implementation Research (CFIR).

CFIR domain	Domain description	Goal	Data source
Outer setting	Characteristics outside the program and program delivery staff (eg, OVP^a^ and PI^b^)	Identify elements external to the program (eg, media) that might shape implementation barriers and facilitators	Community outreach event log
Intervention characteristics	Elements of the program that might cause barriers to implementation and sustainability	Facilitate adaptations of the program and program delivery	PI training tracking
Process	Actions taken during the implementation and delivery of the program that can be defined	Develop strategies that facilitate the implementation of the intervention	Community outreach event log and PI training tracking

^a^OVP: Milwaukee Office of Violence Prevention.

^b^PI: Prevention Institute.

### Ethical Considerations

There is strong evidence that the trauma of community violence impacts those living in these communities, including health effects on youth and adolescents [27]. Importantly, those who are both in the community and engaging in intervention delivery are also greatly impacted [28]. Given these considerations, we will engage community members through focus groups and surveys on trainings to better understand the norms of violence as well as the mental and physical impacts. We will make every effort to support those participating in the study and provide mental health support, as requested. This study has been approved by the institutional review board of Advarra (#0217719). The proposed study will be performed in accordance with the Declaration of Helsinki.

Informed consent will be obtained from all the participants or legal guardians of participants for the community focus groups through signing a digital consent form. Those who attend the focus group will receive a US $40 gift card (participants may choose between Walmart, Amazon, or 2 local Milwaukee vendors). For the community focus groups, the audio of each of the focus groups will be recorded, transcribed, and anonymized. All data are securely stored on the RTI International server, and all electronic files containing identifiable information are password protected, with access limited to study staff. Only aggregate data will be shared with study partners. Focus group recordings will be destroyed as soon as the project is completed.

## Results

This project received funding on September 1, 2020. Prospective study data collection began in the fall of 2021 and will continue through the end of 2023. Data analysis is currently underway, and the first results are expected to be submitted for publication in 2024.

## Discussion

### Expected Findings

This study aims to understand the impacts of a community-centered violence intervention. Our goal is not only to determine the impact of community organizing on community violence prevention but also to determine the feasibility and acceptability of such a strategy. This intervention centers the community, leveraging community member strengths, experiences, knowledge, and wisdom.

A key strength of this study is the engagement with government and community partners, along with the use of existing community infrastructure. Using the ACE|R framework plus community organizing to identify approaches to strengthen the city’s implementation and impacts of the Milwaukee Blueprint for Peace engages resident leaders within an existing policy initiative. We will use our findings to further build the evidence for this approach, including identifying the barriers to and facilitators of implementation as faced by the community and its partners.

The first limitation of this study is the impact of COVID-19 on staffing, recruitment, and data collection. Labor markets have shifted widely during the pandemic; as such, identifying research support staff in Milwaukee may prove difficult. Such impacts are also being seen by other nonprofit organizations and government agencies. Because of our partnerships with the Milwaukee OVP, we may see impacts on its structure and the OVP-led community engagement activities. Recruitment at in-person events is difficult because, despite relaxed COVID-19–related restrictions, there continues to be less in-person interaction; remote events are also challenging as recruiting avenues because of scheduling conflicts and a lower engagement than at in-person events. Because conducting focus groups remotely is difficult, we aim to incorporate in-person focus groups once local Milwaukee officials have determined that it is safe to do so.

Beyond the impacts of COVID-19, an additional limitation with a community-level intervention is the possibility of contamination bias—that is, bleeding of the intervention into control communities. With extensive reporting and regular team meetings with the OVP, the PI, and our community partners, we hope to account for this bias but recognize that it cannot be completely avoided.

### Conclusions

Our hope is that this approach will further strengthen the evidence that not only should community violence be viewed as a public health problem but also that there are ways to address it as such with a community-level approach that centers communities as agents of community transformation. How to implement these types of programs and the methods of measuring their impact remain mixed. The role of resident leaders is pivotal for understanding the effects of the intervention on community violence and the impacts on the leaders themselves in delivering and implementing the intervention. Results from this study will provide a road map for how other communities can implement community organizing within the ACE|R framework and further community violence prevention efforts.

## Appendix

Multimedia Appendix 1 Peer review from the National Center for Injury Prevention and Control, Centers for Disease Control and Prevention (USA).

## Abbreviations

ACE|R: Adverse Community Experiences and Resilience
CFIR: Consolidated Framework for Implementation Research
OVP: Office of Violence Prevention
PI: Prevention Institute

## Data Availability

The data from this study are not publicly available as data collection is ongoing and the results have not been analyzed. Materials for data collection are available from the corresponding author upon reasonable request.
