# Supplementary material for: Resilience-Informed Community Violence Prevention and Community Organizing Strategies for Implementation: Protocol for a Hybrid Type 1 Implementation-Effectiveness Trial
Source: JMIR Res Protoc. 2023 Nov 7;12:e50444. doi: 10.2196/50444 (PMC10664006; doi:10.2196/50444)
Supplement: Multimedia Appendix 1 [file resprot_v12i1e50444_app1.pdf]

SUMMARY STATEMENT

PROGRAM CONTACT: (Privileged Communication) *Release Date:* 07/31/2020

Revised Date:

Application Number: 1 R01 CE003191-01

Principal Investigators (Listed Alphabetically):

GRAHAM, PHILLIP W (Contact)
YAROS, ANNA

Applicant Organization: RESEARCH TRIANGLE INSTITUTE

Review Group: ZCE1 KFJ (01)
National Center for Injury Prevention Special Emphasis Panel
CE20-003 - Research Grants for Preventing Violence and Violence Related Injury

Meeting Date: 04/01/2020
RFA/PA: CE20-003
Council: MAY 2020
Requested Start: 09/01/2020

Project Title: Effectiveness of Adverse Community Experiences and Resilience (ACER) and Community Organizing for Preventing Youth Violence and ACEs.
SRG Action: Impact Score:32

Human Subjects: 30-Human subjects involved - Certified, no SRG concerns
Animal Subjects: 10-No live vertebrate animals involved for competing appl.
Gender: 1A-Both genders, scientifically acceptable
Minority: 1A-Minorities and non-minorities, scientifically acceptable
Age: 1A-Children, Adults, Older Adults, scientifically acceptable
Clinical Research - not NIH-defined Phase III Trial

Project Year	Direct Costs Requested	Estimated Total Cost
1	243,220	348,753
2	244,211	350,174
3	242,828	348,191
TOTAL	730,259	1,047,119

1R01CE003191-01 GRAHAM, PHILLIP

BUDGET MODIFICATIONS RECOMMENDED

RESUME AND SUMMARY OF DISCUSSION: This application, by a seasoned Principal Investigator (PI), proposes to evaluate the Prevention Institute's implementation of the Adverse Community Experiences and Resilience (ACE|R) community-level violence prevention framework, responding to Objective 1 of the Notice of Funding Opportunity (NOFO). The specific aims are to: 1) retroactively measure the effectiveness of the ACE|R framework implemented in Milwaukee on child abuse and neglect (CAN) and youth violence (YV) using an idiographic clinical trial approach; 2) prospectively test the comparative effectiveness of the ACE|R framework/Milwaukee Blueprint for Peace along with community organizing in four priority communities; and 3) use a Hybrid Type 1 effectiveness-implementation design to explore the barriers and facilitators to implementing community organizing within the ACE|R framework in a large metropolitan area of Milwaukee to inform future implementation efforts by determining strategies to address implementation barriers arising from (a) the intervention characteristics, (b) outer and inner setting factors that shape implementation barriers and facilitators, (c) individual characteristics among stakeholders that influence implementation barriers and facilitators, and (d) strategies that facilitate implementation of community organizing within the ACE|R framework. Reviewers determined this proposal to have high potential for public health impact.

In terms of significance, a major strength of this application is that it directly responds to Objective 1 of the NOFO, targeting a community-level approach to reducing rates of child maltreatment (CM) and YV. The study will assess effective strategies for sustaining change, particularly with regards to barriers and facilitators to the interventions that follow from the ACE|R framework. ACE|R is a community-driven primary prevention framework that promotes a data-driven approach in community strategies that engage the community in identifying risk and protective factors in three areas: social and cultural environment, physical environment, and economic environment. With respect to the investigators, the project team is from Research Triangle Institute International (RTI). RTI was the original research group in Research Triangle Park, NC with three founding universities (Duke University, University of North Carolina-Chapel Hill, and North Carolina State University). Having grown into an international research group, they have extensive staff and expertise and have worked with these types of complex projects. The investigators assembled for this project have a vast amount of relevant experience. Plans are outlined to build upon existing collaborative relationships, many of which extend from the four-year project period of the Blueprint for Peace. Another partner is the City of Milwaukee Office of Violence Prevention, which will be providing city-level data for some of the analyses and from whom appropriate letters of support (LOS) are provided. This group also has extensive experience working with the community. Innovative aspects of the proposed study include the community-driven approach and the hybrid effectiveness-implementation design, which promises to examine barriers and facilitators to implementation and has the potential to move the field forward.

Regarding the approach, the aims are well-aligned with the goals of the NOFO. The overall proposal is clear and detailed in terms of its plans, objectives, activities, and the logic model. A number of outcomes are listed in the logic model that are aligned with the data sources to be utilized, including: community-level reduction in childhood adverse experiences (ACEs), CAN, community-level YV, confirmed and suspected child abuse, violence-related injuries, Child Protective Services (CPS) referrals, CM reports, calls for emergency and non-emergency service numbers, arrests due to CM and violence, suspensions and expulsions from school due to fighting, community ACEs, proportion of single parent households, proportion of female headed households, incarceration rates, mental illness prevalence, substance abuse prevalence, renter occupied housing, average household income, internet access, education level, and employment. The data sources and data identified with which to assess the more violent and ACEs-related outcomes should be useful in terms of assessing change and community-level interventions. The community-driven approach is a major strength of the proposed

study. The ACE|R approach itself is well-grounded in theory, and the proposed project builds upon four years of work in Milwaukee, WI. The investigators, along with the Prevention Institute, have worked with the community group in the development of the Milwaukee Blueprint for Peace. The applicant is now proposing to retrospectively evaluate the effectiveness of the framework's implementation in Milwaukee, WI on CAN and YV, which will then be taken forward to integrate a community organizing and mobilizing approach. The community partner in Milwaukee, WI will continue to provide and expand technical training to the community members to determine whether the strategies developed can be implemented in the four priority communities. The novel Hybrid Type 1 effectiveness implementation design to be employed in the comparison communities to identify barriers and facilitators to implementation is also well-described. Additionally, incentives are planned for the qualitative component.

Weaknesses also were identified. Regarding significance, there are some limitations with regards to the potential generalizability of the findings to communities outside of Milwaukee, WI, which is not addressed in the application. With respect to the investigators, over 600 qualitative interviews with community members are proposed. The project team, however, appears to have limited qualitative research experience. The individual identified that is responsible for this task is a public health analyst for surveillance data who does not have any publications in qualitative research. Additionally, it is not clear whether any of the three team members from the Prevention Institute are physically located in Milwaukee, WI. A letter of support is provided from the City of Milwaukee Office of Violence Prevention with which the investigators are partnering, however, the particular staff from that office is not included in the proposal as a team member. This, however, is tempered by the proposed study having a strong focus on being community-driven, the emphasis on building capacity with community leadership, and the fact that the project truly is being placed in the hands of the community.

In terms of the approach, it is unclear whether the qualitative expertise and resources identified will be sufficient for the considerable amount of qualitative data that are likely to be generated. Clear plans are not delineated for how these data will be analyzed and managed or who will be responsible for this effort. This is a potential shortcoming, given that analysis of this large amount of data is going to require a major amount of effort. Seemingly, there is a lot of dependence on retrospective administrative data; however, there is insufficient discussion about the inherent limitations of this type of data. In terms of fidelity, it is not clear that it will be possible to adequately document how well some of the programs have been implemented over the last three years to ensure that the retrospective data are clean. There was a lack of control in the previously completed community-level program in terms of fidelity and dosage, and it is not clear how this would relate to the proposed project's outcomes. Additionally, there was a comment about the limitations on how quickly the available administrative data are ready to use. However, a statement is made that the investigators are working more closely with Milwaukee, WI to focus on real-time available data on a monthly basis. Beyond this, there is little discussion about the potential limitations of the administrative data. It is unclear whether the administrative data will lend itself to clear measurement with all of the outcome variables mentioned, particularly with regards to the demonstration of impact and change at the community-level. Nevertheless, the investigators have been receiving these data for a long period of time and they have an existing relationship with the city of Milwaukee, WI. The power analysis does not clearly center upon a primary endpoint, and a power analysis is not provided for the proposed idiographic clinical trials (ICTs) analysis. The translation plan is somewhat limited in terms of broader dissemination to policymakers and to the public. Also, regarding the environment, it is difficult to assess the Prevention Institute's facilities and resources that will be dedicated to the proposed study.

Protection of human subjects is acceptable. Inclusion is acceptable in terms of gender, minority groups, and age. Animal welfare, biohazards, and dual use research are not applicable. Budget modifications are recommended, as 60% of the funds are allocated to a sub-awardee. An acceptable resource

sharing plan/data management plan (DMP) is provided. Overall, this is an excellent application that is very strong with only some minor weaknesses.

DESCRIPTION (provided by applicant): Violence is one of several Adverse Childhood Experiences (ACEs) that can significantly impact children's future physical and behavioral health. Different types of violence against youth, such as child abuse and neglect and community youth violence, often share the same risk and protective factors. Community disadvantage and disorganization are risk factors associated with community trauma and violence, which can be addressed by community-level primary prevention strategies that emphasize community resilience through community organizing. This study will evaluate Prevention Institute's implementation of the Adverse Community Experiences and Resilience (ACE|R) community-level violence prevention framework, responding to Objective 1: Effectiveness research to determine which community- or societal-level strategies effectively prevent multiple forms of violence and other ACEs that impact children and youth. More specifically, it has three aims: (1) retroactively measure the effectiveness of the ACE|R framework implemented in Milwaukee on child abuse and neglect and youth violence using an idiographic clinical trial approach; (2) prospectively test the comparative effectiveness of the ACE|R framework/Milwaukee Blueprint for Peace with community organizing in four Priority Communities; and (3) use a Hybrid Type 1 effectiveness-implementation design to explore the barriers and facilitators to implementing community organizing within the ACE|R framework in a large metropolitan area (Milwaukee) to inform future implementation efforts by determining strategies to address implementation barriers arising from (a) the intervention characteristics, (b) outer and inner setting factors that shape implementation barriers and facilitators, (c) individual characteristics among stakeholders that influence implementation barriers and facilitators, and (d) strategies that facilitate implementation of community organizing within the ACE|R framework. By addressing several factors associated with community trauma and violence, this framework has the potential to prevent child abuse, child neglect, and youth violence. Findings will expand research evidence for the facilitation of community resilience efforts and contribute valuable insight regarding efficacious, practice-based violence prevention programming.

PUBLIC HEALTH RELEVANCE: This study will evaluate the Adverse Community Experiences and Resilience (ACE|R) framework for community-level violence prevention through economic, social, and place-based strategies to heal trauma in communities. We will test (a) whether this process has reduced rates of child abuse and neglect and youth violence over the past 4 years of its implementation in the Milwaukee Blueprint for Peace and (b) the benefits of adding community organizing focused on engaging and mobilizing community members to support strategies to prevent violence. Our findings will provide insight about the best ways to help neighborhoods and cities fight child abuse/neglect and youth violence.

CRITIQUE 1

Significance:	1
Investigator(s):	2
Innovation:	1
Approach:	2
Environment:	1

Overall Impact:
Strengths
- If the aims are achieved, the proposed project will identify an effective community-level approach to reducing rates of child maltreatment and youth violence, as well as an effective strategy for sustaining community change.
- The project will also provide valuable information on potential barriers and facilitators to inform implementation of this approach across communities.

Weaknesses

- The limitations on the generalizability of findings to communities outside Milwaukee, WI are not addressed.

1. Significance:
Strengths
- The proposed project aims to identify an effective community-level, community-driven approach to preventing child maltreatment and violence, as well as other adverse childhood experiences, and to examine its comparative effectiveness across communities.
- The project further aims to evaluate the effectiveness of community organizing and mobilizing as an effective strategy for sustaining positive change.

Weaknesses:
- The limitations on the generalizability of findings to communities outside Milwaukee, WI are not addressed.

2. Investigator(s):
Strengths
- The project team, including researchers at RTI International; the technical training experts at the Prevention Institute, whose role includes providing technical assistance to participating communities; and the City of Milwaukee Office of Violence Prevention, which will facilitate implementation and retrieval of community-level data, is well-suited to conduct the work proposed.
- The principal investigator has more than 20 years of experience conducting community-based research and evaluation on youth violence, including multiple federal-funded projects, and will be supported by a strong research team, including individuals with extensive experience managing and coordinating projects of this scale.
- Both the principal investigator and the project director also have expertise related to the role of culture and diversity in prevention research.
- The project leverages collaborative relationships built throughout the four-year implementation of the Milwaukee Blueprint for Peace, which is based on the Adverse Community Experiences and Resilience (ACE|R) framework, which is the subject of evaluation in the proposed study.

Weaknesses
- The specific personnel at the Prevention Institute and the City of Milwaukee Office of Violence Prevention who will be involved with the project were not identified.

3. Innovation:
Strengths
- The project proposes to evaluate the effectiveness of community organizing and mobilization as a novel approach to building and sustaining community resilience by addressing community disadvantage and disorganization, which are risk factors associated with community trauma and violence.
- The project employs a novel hybrid-effectiveness implementation design with the goal of maximizing communities' ability to sustain intervention strategies shown to be effective by moving beyond describing standard implementation elements, such as dose and fidelity, to closing the implementation gap.

Weaknesses
- No weaknesses are noted.

4. Approach:
Strengths
- The aims of the proposed project are directly aligned with Objective 1 of the NOFO. The project has the potential to identify an effective community-level strategy for preventing child maltreatment and youth violence, as well as other adverse childhood experiences.

- The theoretical and evidentiary base for the Adverse Community Experiences and Resilience (ACE|R) framework, as well as the addition of community organizing to enhance sustainability by building resilience and addressing risk factors associated with community risk for violence, are well-developed.
- The proposal anticipates and provides a clear, detailed plan for activities required to complete the project successfully, from implementation through data analysis and dissemination. Also, there is a clear logic model.
- The project methodology and plan for data analysis is rigorous and empirically sound. Also, mixed methods are employed.

Weaknesses

- The plan for management and analysis of qualitative data was not well-developed.

5. Environment:

Strengths

- RTI International is a well-established independent, nonprofit research organization with an expansive research infrastructure to support complex research projects in partnership with a wide range of entities, including government, business, and universities.
- The project leverages existing collaborations among the project partners, including a five-year partnership between the Prevention Institute and the City of Milwaukee to implement the Blueprint for Peace.
- There is ample evidence of a clear delineation of the specific roles and responsibilities of the researchers and other project partners, as well as their understanding of these roles and responsibilities.

Weaknesses

- No weaknesses are noted.

Protections for Human Subjects
Acceptable Risks and/or Adequate Protection

- A thorough plan was provided.

Data and Safety Monitoring Plan (Applicable for Clinical Trials Only)
Not Applicable

Inclusion of Women, Minorites, and Age (Applicable Only for Human Subjects Research)
G1A=Includes both genders, acceptable
M1A=Includes minorities and non-minorities, acceptable
C1A=Includes children, adults, and older adults, acceptable

- The focus on adult members of participating communities is appropriate to the focus of this research.

Vertebrate Animals
Not Applicable (No Vertebrate Animals)

Biohazards
Not Applicable (No Biohazards)

Dual Use Research of Concern
Not Applicable

Budget and Period of Support
Recommend as Requested

Resource Sharing Plans
Acceptable

Data Management Plan (DMP)
Acceptable
- The Data Management Plan is applicable, present, appropriate.

CRITIQUE 2

Significance:	1
Investigator(s):	2
Innovation:	1
Approach:	3
Environment:	2

Overall Impact
Strengths
- The project directly addresses the NOFO as well as the lack of evidence on the effectiveness of community-level strategies for the prevention of ACEs (specifically child abuse and neglect and youth violence). The investigators propose to examine the effectiveness of community organizing and mobilization in changing community-level risk factors to reduce child maltreatment and youth violence within urban communities.
- There is a strong potential for the proposed project to have a positive impact on multiple forms of youth violence at a community level.
- The proposed project builds on a current initiative and current partnerships.

Weaknesses
- A minor weakness is the lack of a community partner (City of Milwaukee Office of Violence Prevention) on the investigative team. However, the investigators have worked in these communities for the past four years and the Prevention Institute has experience working with communities.

1. Significance:
Strengths
- The project directly addresses the lack of evidence on the effectiveness of community-level strategies for the prevention of ACEs (specifically child abuse and neglect and youth violence).
- The proposed intervention activities, using the Adverse Community Experiences and Resilience (ACE|R) framework, have the potential to reduce the level of child abuse and neglect and youth violence at the community level. The ACE|R framework clearly focuses on risk and protective factors at the community and societal level.
- If successful, the proposed research will enhance our understanding of the effectiveness of community organizing and mobilization in changing community-level risk factors to reduce child maltreatment and youth violence within urban communities.

Weaknesses
- No weaknesses are noted.

2. Investigator(s):
Strengths
- The PI Dr. Graham has a long history of conducting rigorous, community-based research and evaluating the effectiveness of youth violence prevention interventions in real-world and community-based settings and brings expertise in program evaluation, intervention adaptation, effectiveness trials, social determinants, implementation research, community-based and community-level research among diverse populations and settings, and implementation and prevention science.

- The PI Dr. Yaros, an earlier career investigator, also brings expertise in mental health services in schools, school safety and discipline, parental aggression (e.g., child maltreatment), ACEs research, and evaluation of mental health services, and is well suited to lead the project with PI Dr. Graham.
- The Co-PIs both have extensive experience leading multi-site, multi-investigator evaluation and research teams.
- The Prevention Institute TTA team members have a history of providing technical assistance and support for ACE|R framework activities in Milwaukee, WI. The Prevention Institute has been working with the City of Milwaukee Office of Violence Prevention since 2016 to implement the ACE|R framework through the citywide Blueprint for Peace.
- The investigators have a history of collaboration and are building on an ongoing community initiative.

Weaknesses

- A minor weakness is that the City of Milwaukee Office of Violence Prevention is described as a partner but is not included as part of the investigative team.

3. Innovation:

Strengths

- The ACE|R framework is innovative.
- The incorporation of community organizing to the current ACE|R framework and Blueprint for Peace initiative is innovative.
- The hybrid effectiveness-implementation design is innovative.

Weaknesses

- No weaknesses are noted.

4. Approach:

Strengths

- The investigators clearly address primary outcomes 1 and 2 of the NOFO, including: 1) to determine which community-level or societal-level strategies effectively prevent multiple forms of violence and other ACEs that impact children and youth; and 2) evaluate strategies that enhance protective factors.
- The investigators propose a retrospective and prospective design that builds on current and previous work (work that begun in 2016) and enables the ability to examine long-term effectiveness of a community-level prevention strategy.
- The proposed project leverages other activities and funding in the community, including BUILD funding in one of the community sites.
- The proposed hybrid type 1 effectiveness-implementation design is appropriate for the proposed study. The evaluation includes an extensive evaluation of the implementation process guided by the Consolidated Framework for Implementation Research.

Weaknesses

- It is unclear what outcome was used to determine power, as the power analysis is not specific to an outcome.
- A minor weakness is the application includes a limited translation plan. The application includes discussion of creating an implementation manual and predominantly academic publications and presentations. It lacks detail on broader dissemination to the public and policy makers.

5. Environment:

Strengths

- Letters of support are included.

Weaknesses

- No weaknesses are noted.

Protections for Human Subjects
Acceptable Risks and/or Adequate Protection

Data and Safety Monitoring Plan (Applicable for Clinical Trials Only)
Not Applicable

Inclusion of Women, Minorites, and Age (Applicable Only for Human Subjects Research)
G1A=Includes both genders, acceptable
M1A=Includes minorities and non-minorities, acceptable
C1A=Includes children, adults, and older adults, acceptable

- Acceptable for the proposed study.

Vertebrate Animals
Not Applicable (No Vertebrate Animals)

Biohazards
Not Applicable (No Biohazards)

Dual Use Research of Concern
Not Applicable

Budget and Period of Support
Recommend as Requested

Resource Sharing Plans
Acceptable

Data Management Plan (DMP)
Acceptable

CRITIQUE 3

Significance:	2
Investigator(s):	5
Innovation:	3
Approach:	4
Environment:	3

Overall Impact
Strengths

- This application proposes to evaluate the effectiveness of the Adverse Community Experiences and Resilience (ACE|R) program, a community-level prevention framework to address child abuse and neglect and youth violence. It includes three major aims to measure program effectiveness in one city (Milwaukee, WI), prospective testing of this program, and to explore barriers and facilitators.
- This application directly addresses Objective 1 of the NOFO by proposing both effectiveness and implementation research and building upon previous prevention programming with additional community-level approaches.

Weaknesses

- A minor weakness relates to the lack of translatability and generalizability of the findings for communities that differ economically, racially, urbanicity, and in size from Milwaukee, WI.

1. Significance:

Strengths

- This application proposes to assess the impact of the Adverse Community Experiences and Resilience (ACE|R) framework on changing the social-cultural, physical, and economic environments in a large metropolitan city, Milwaukee, WI. It focuses on both risk factors and resilience strategies through community-level action to prevent child abuse, neglect, and youth violence.
- The application outlines the significant rates of child abuse, neglect, and youth violence within the Milwaukee community that demonstrates a community in high need of community-level services.

Weaknesses

- No weaknesses are noted.

2. Investigator(s):

Strengths

- The Principal Investigators Graham and Yaros have extensive experience in prevention science and research, community-based research, research record in violence prevention, and significant experience in the management of large-scale, cross-site grants. A strong rationale is provided for multiple PIs.
- Co-I Ridenour has extensive quantitative research analytic training appropriate for the proposed analyses.
- Ms. Embry has appropriate experience as a data collection manager.
- The application combines the expertise of RTI professionals with the Prevention Institute who will provide technical and training assistance to the Blueprint for Peace and City of Milwaukee Office of Violence Prevention.

Weaknesses

- Co-I Cance has appropriate experience as a public health analyst for surveillance data but is described as the lead qualitative data analyst for which she has no published experience.

3. Innovation:

Strengths

- The application states the ACE|R framework is to be evaluated using longitudinal surveillance data and Child Protective Services report which are likely to represent the outcomes (child abuse, neglect, youth violence) accurately.
- This application capitalizes on evaluating a preexisting community-level violence prevention program through a hybrid effectiveness-implementation design, merging the importance of scientific data with currently occurring public health action.

Weaknesses

- A minor weakness is the lack of control in the implementation of a previously completed community-level prevention program that may have lacked in fidelity and dosage (or lacks adequate records of these). This limitation may reduce the ability to adequately translate the minimum level of effectiveness for positive outcomes.

4. Approach:

Strengths

- This application builds on previous efforts to analyze retrospective community level data while completing community planning to mobilize four priority communities to receive additional community support through technical and training assistance, and continuing business as usual (ACE|R) in six other priority communities.
- The proposed improvements in outcomes include strengthening communities, reducing violence and abuse, and increasing social organization, which directly addresses Objective 1.

- The approach includes longitudinal qualitative methods of archival materials and stakeholder interviews to explore barriers and facilitators which may likely provide relevant information to improve implementation of community-level interventions.

Weaknesses

- With a focus on surveillance data, unknown and unreported incidents of violence, abuse, and neglect will not be captured, which may be important for seeing change occurring at a smaller level.
- There are many proposed qualitative interviews (600) that may result in a large amount of data that may be unwieldy to analyze, particularly without a qualitative expert.
- The power analysis includes appropriate information but is not explicitly specific to the proposed idiographic clinical trial analysis.

5. Environment:

Strengths

- The proposed application is to be completed at Research Triangle Institute (RTI) International which has a strong record of research accomplishment, substantial organization support, and a quality research operations center.
- Several large organizations (RTI, Prevention Institute, City of Milwaukee Office of Violence Prevention) can combine efforts to evaluate previous community-level interventions, while introducing and evaluating new components, building on the capital of current efforts.

Weaknesses

- A sub-awardee is the Prevention Institute and is budgeted at 60% of the award. The facilities and resource of Prevention Institute are only minimally addressed in the application.

Protections for Human Subjects
Acceptable Risks and/or Adequate Protection

- The application clearly outlines the procedures in place to minimize risk, maintain confidentiality, and appropriate planned research procedures for human subjects research.

Data and Safety Monitoring Plan (Applicable for Clinical Trials Only)
Not Applicable

Inclusion of Women, Minorites, and Age (Applicable Only for Human Subjects Research)
G1A=Includes both genders, acceptable
M1A=Includes minorities and non-minorities, acceptable
C1A=Includes children, adults, and older adults, acceptable

- The proposed inclusion is unknown because they are not selecting, recruiting, or limiting by gender/sex, race/ethnicity, and age, but are using archival data from Child Protective Services and surveillance data. Qualitative interviews with adults are based on demographics of community stakeholders and interventionists.

Vertebrate Animals
Not Applicable (No Vertebrate Animals)

Biohazards
Not Applicable (No Biohazards)

Dual Use Research of Concern
Not Applicable

Budget and Period of Support
Budget Modifications Recommended

- A large portion of the budget (60%) is intended for a sub-awardee.

Resource Sharing Plans
Acceptable

Data Management Plan (DMP)
Acceptable

- The data management plan includes provisions for maintaining confidentiality of qualitative data, as well as for sharing quantitative data in appropriate formats.

THE FOLLOWING SECTIONS WERE PREPARED BY THE SCIENTIFIC REVIEW OFFICER TO SUMMARIZE THE OUTCOME OF DISCUSSIONS OF THE REVIEW COMMITTEE, OR REVIEWERS' WRITTEN CRITIQUES, ON THE FOLLOWING ISSUES:

PROTECTION OF HUMAN SUBJECTS: ACCEPTABLE

INCLUSION OF WOMEN PLAN: ACCEPTABLE
Includes both genders and is scientifically acceptable.

INCLUSION OF MINORITIES PLAN: ACCEPTABLE
Includes minorities and non-minorities and is scientifically acceptable.

INCLUSION OF AGE PLAN: ACCEPTABLE
Includes children, adults, and older adults and is scientifically acceptable.

COMMITTEE BUDGET RECOMMENDATIONS: The committee noted that a large portion of the budget (60%) is intended for a sub-awardee, which warrants a detailed explanation as to why a large portion of the funds are allocated to the sub-awardee.

COMMITTEE RESOURCE SHARING/ DATA MANAGEMENT PLAN RECOMMENDATIONS:
The resource sharing/data management plans are acceptable.

MEETING ROSTER

National Center for Injury Prevention Special Emphasis Panel
NATIONAL CENTER FOR INJURY PREVENTION AND CONTROL
CE20-003 - Research Grants for Preventing Violence and Violence Related Injury

ZCE1 KFJ (01)
04/01/2020 - 04/02/2020

CHAIRPERSON(S)

CUDDEBACK, GARY S, PHD
PROFESSOR
SCHOOL OF SOCIAL WORK
UNIVERSITY OF NORTH CAROLINA
CHAPEL HILL, NC 27599

MEMBERS

BAKER, CHARLENE ,PHD
PROFESSOR & CHAIR
DEPARTMENT OF PSYCHOLOGY
UNIVERSITY OF HAWAII
HONOLULU, HI 96825

BLOOM, TINA L, PHD
ASSOCIATE PROFESSOR
SCHOOL OF NURSING
UNIVERSITY OF MISSOURI
COLUMBIA, MO 65211

DEBNAM, KATRINA J, PHD
ASSISTANT PROFESSOR
SCHOOL OF NURSING
UNIVERSITY OF VIRGINIA
CHARLOTTESVILLE, VA 22908

FAGAN, ABIGAIL ANNE, PHD
PROFESSOR
DEPARTMENT OF SOCIOLOGY AND CRIMINOLOGY & LAW
UNIVERSITY OF FLORIDA
GAINESVILLE , FL 32611

GONCY, ELIZABETH A, PHD
ASSISTANT PROFESSOR
DEPARTMENT OF PSYCHOLOGY
CLEVELAND STATE UNIVERSITY
CLEVELAND, OH 44115

OSTROV, JAMIE M, PHD
PROFESSOR
DEPARTMENT OF PSYCHOLOGY
STATE UNIVERSITY OF NEW YORK-BUFFALO
BUFFALO, NY 14260

PORTWOOD, SHARON, PHD, JD
PROFESSOR
DEPARTMENT OF PUBLIC HEALTH SCIENCES
UNIVERSITY OF NORTH CAROLINA AT CHARLOTTE
CHARLOTTE, NC 28223

RENNER, LYNETTE MICHELLE, PHD
ASSOICATE PROFESSOR
SCHOOL OF SOCIAL WORK
UNIVERSITY OF MINNESOTA
ST. PAUL, MN 55108

SHEEHAN, KAREN MAY, MD
ATTENDING PHYSICIAN
PEDIATRIC EMERGENCY MEDICINE
LURIE CHILDREN’S HOSPITAL OF CHICAGO
CHICAGO, IL 60611

STODDARD, SARAH A, PHD
ASSISTANT PROFESSOR
SCHOOL OF NURSING
UNIVERSITY OF MICHIGAN
ANN ARBOR, MI 48109

WILDEMAN, CHRISTOPHER, PHD
PROFESSOR
DEPARTMENT OF POLICY ANALYSIS & MANAGEMENT
CORNELL UNIVERSITY
ITHACA, NY 14853

WRIGHT, EMILY M., PHD
PROFESSOR
SCHOOL OF CRIMINOLOGY AND CRIMINAL JUSTICE
UNIVERSITY OF NEBRASKA OMAHA
OMAHA, NE 68182

SCIENTIFIC REVIEW OFFICER

LEEKS, KIMBERLY D, PHD
SCIENTIFIC REVIEW OFFICER
NATIONAL CENTER FOR INJURY PREVENTION AND
CONTROL
NATIONAL CENTER FOR ENVIORNMENTAL HEALTH/ATSDR
EXTRAMURAL RESEARCH PROGRAM OFFICE
CENTERS FOR DISEASE CONTROL AND PREVENTION
ATLANTA, GA 30341

EXTRAMURAL SUPPORT ASSISTANT

ROBERTS, KENNETH H
GRANTS TECHNICAL ASSISTANT
NATIONAL CENTER FOR INJURY PREVENTION & CONTROL
NATIONAL CENTER FOR ENVIRONMENTAL HEALTH/ATSDR
EXTRAMURAL RESEARCH PROGRAM OFFICE
CENTERS FOR DISEASE CONTROL & PREVENTION
ATLANTA, GA 30341

Consultants are required to absent themselves from the room during the review of any application if their presence would constitute or appear to constitute a conflict of interest.
